# Supplementary material for: End Sequence Analysis Toolkit (ESAT) expands the extractable information from single-cell RNA-seq data
Source: Genome Res. 2016 Oct;26(10):1397–410. doi: 10.1101/gr.207902.116 (PMC5052061; doi:10.1101/gr.207902.116)
Supplement: Supplemental Material [file supp_gr.207902.116_Supp_Materials.tar.gz › ESAT-esat_v0.1/doc/html/classbroad_1_1core_1_1motif_1_1_sequence_motif.html]

ESAT: broad.core.motif.SequenceMotif Class Reference


ESAT


- Main Page
- Related Pages
- Packages
- Classes
- Files

- Class List
- Class Index
- Class Hierarchy
- Class Members

All Classes Namespaces Files Functions Variables Enumerator Pages

- broad
- core
- motif
- SequenceMotif

Public Member Functions |
Static Public Member Functions |
Static Public Attributes |
Protected Member Functions |
List of all members

broad.core.motif.SequenceMotif Class Reference

Inheritance diagram for broad.core.motif.SequenceMotif:

[legend]

Collaboration diagram for broad.core.motif.SequenceMotif:

[legend]

|  |  |
| --- | --- |
| Public Member Functions | |
|  | SequenceMotif (Pattern motif) |
|  | |
|  | SequenceMotif (String corePattern, String identifier) throws SearchException |
|  | |
|  | SequenceMotif (String corePattern) throws SearchException |
|  | |
|  | SequenceMotif (String corePattern, int minsuccessive) throws SearchException |
|  | |
|  | SequenceMotif (String corePattern, int minsuccessive, String identifier) throws SearchException |
|  | |
| String | getId () |
|  | |
| String | getConsensusSequence () |
|  | |
| int | getNumPossibleKmers () |
|  | |
| Pattern | getMotif () |
|  | |
| List< SequenceRegion > | match (Sequence seq) |
|  | |
| boolean | equals (Object o) |
|  | |
| String | toString () |
|  | |
| int | hashCode () |
|  | |
| List< SequenceRegion > | match (Sequence geneSeq, List< SequenceRegion > list, int length) |
|  | |

|  |  |
| --- | --- |
| Static Public Member Functions | |
| static int | numPossibleKmers (String consensusSequence) |
|  | |
| static void | main (String[] args) throws NumberFormatException, SearchException, IOException |
|  | |

|  |  |
| --- | --- |
| Static Public Attributes | |
| static String | USAGE |
|  | |

|  |  |
| --- | --- |
| Protected Member Functions | |
| void | setMotif (Pattern motif) |
|  | |
| Pattern[] | getCoreMotifs () |
|  | |

## Constructor & Destructor Documentation

|  |  |  |  |  |  |
| --- | --- | --- | --- | --- | --- |
| broad.core.motif.SequenceMotif.SequenceMotif | ( | Pattern | *motif* | ) |  |

Here is the caller graph for this function:

|  |  |  |  |
| --- | --- | --- | --- |
| broad.core.motif.SequenceMotif.SequenceMotif | ( | String | *corePattern*, |
|  |  | String | *identifier* |
|  | ) |  | throws SearchException |

|  |  |  |  |  |  |
| --- | --- | --- | --- | --- | --- |
| broad.core.motif.SequenceMotif.SequenceMotif | ( | String | *corePattern* | ) | throws SearchException |

|  |  |  |  |
| --- | --- | --- | --- |
| broad.core.motif.SequenceMotif.SequenceMotif | ( | String | *corePattern*, |
|  |  | int | *minsuccessive* |
|  | ) |  | throws SearchException |

|  |  |  |  |
| --- | --- | --- | --- |
| broad.core.motif.SequenceMotif.SequenceMotif | ( | String | *corePattern*, |
|  |  | int | *minsuccessive*, |
|  |  | String | *identifier* |
|  | ) |  | throws SearchException |

## Member Function Documentation

|  |  |  |  |  |  |
| --- | --- | --- | --- | --- | --- |
| boolean broad.core.motif.SequenceMotif.equals | ( | Object | *o* | ) |  |

Here is the call graph for this function:

Here is the caller graph for this function:

|  |  |  |  |  |
| --- | --- | --- | --- | --- |
| String broad.core.motif.SequenceMotif.getConsensusSequence | ( |  | ) |  |

|  |  |  |  |  |
| --- | --- | --- | --- | --- |
| Pattern [] broad.core.motif.SequenceMotif.getCoreMotifs | ( |  | ) |  |

protected

|  |  |  |  |  |
| --- | --- | --- | --- | --- |
| String broad.core.motif.SequenceMotif.getId | ( |  | ) |  |

|  |  |  |  |  |
| --- | --- | --- | --- | --- |
| Pattern broad.core.motif.SequenceMotif.getMotif | ( |  | ) |  |

|  |  |  |  |  |
| --- | --- | --- | --- | --- |
| int broad.core.motif.SequenceMotif.getNumPossibleKmers | ( |  | ) |  |

Here is the call graph for this function:

|  |  |  |  |  |
| --- | --- | --- | --- | --- |
| int broad.core.motif.SequenceMotif.hashCode | ( |  | ) |  |

Here is the call graph for this function:

Here is the caller graph for this function:

|  |  |  |  |  |  |
| --- | --- | --- | --- | --- | --- |
| static void broad.core.motif.SequenceMotif.main | ( | String[] | *args* | ) | throws NumberFormatException, SearchException, IOException |

static

Parameters
:   |  |  |
    | --- | --- |
    | args |  |

Exceptions
:   |  |  |
    | --- | --- |
    | SearchException |  |
    | NumberFormatException |  |
    | IOException |  |

Here is the call graph for this function:

|  |  |  |  |  |  |
| --- | --- | --- | --- | --- | --- |
| List<SequenceRegion> broad.core.motif.SequenceMotif.match | ( | Sequence | *seq* | ) |  |

Here is the caller graph for this function:

|  |  |  |  |
| --- | --- | --- | --- |
| List<SequenceRegion> broad.core.motif.SequenceMotif.match | ( | Sequence | *geneSeq*, |
|  |  | List< SequenceRegion > | *list*, |
|  |  | int | *length* |
|  | ) |  |  |

Here is the call graph for this function:

|  |  |  |  |  |  |
| --- | --- | --- | --- | --- | --- |
| static int broad.core.motif.SequenceMotif.numPossibleKmers | ( | String | *consensusSequence* | ) |  |

static

Here is the caller graph for this function:

|  |  |  |  |  |  |
| --- | --- | --- | --- | --- | --- |
| void broad.core.motif.SequenceMotif.setMotif | ( | Pattern | *motif* | ) |  |

protected

|  |  |  |  |  |
| --- | --- | --- | --- | --- |
| String broad.core.motif.SequenceMotif.toString | ( |  | ) |  |

Here is the caller graph for this function:

## Member Data Documentation

String broad.core.motif.SequenceMotif.USAGE

static

**Initial value:**

= "Usage: SequenceMotif TASK=<task\_num> <task\_args>\n" +

"\tTasks:\n" +

"\t\t1. Get list of motif matches: IN=<fasta sequence> MOTIF=<Core Motif e.g. YRNRY> SUSCCESSIVE=<number of consecutive core motifs>\n"

---

The documentation for this class was generated from the following file:

- java/broad/core/motif/SequenceMotif.java


---

Generated on Fri Aug 15 2014 15:10:54 for ESAT by  

 1.8.7
